# Correlation between insulin resistance and the rate of neutrophils-lymphocytes, monocytes-lymphocytes, platelets-lymphocytes in type 2 diabetic patients

**DOI:** 10.1186/s12902-024-01564-x

**Published:** 2024-03-25

**Authors:** Yuanyuan Zhang, Huaizhen Liu

**Affiliations:** Endocrinology Department, Senile Disease Center, The First Affiliated Hospital of Anhui, University of Traditional Chinese Medicine, 117 Meishan Road, 230009 Hefei, Anhui China

**Keywords:** Type 2 diabetes mellitus, Insulin resistance, Neutrophils-lymphocytes rate, Monocytes-lymphocytes rate, Platelets-lymphocytes rate

## Abstract

**Background:**

Insulin resistance (IR) was a prominent feature commonly observed in individuals with type 2 diabetes mellitus (T2DM). T2DM Individuals often exhibited a concomitant presence of low-grade chronic inflammation. In this study conducted retrospectively, the aim was to investigate the connection between neutrophils-lymphocytes rate (NLR), monocytes-lymphocytes rate (MLR), platelets-lymphocytes rate (PLR) and IR, specifically among individuals with T2DM.

**Method:**

This study encompassed a cohort of 405 individuals diagnosed with T2DM, comprising cases from January 2021 to November 2022. On the basis of whether there was IR or not, these sufferers were categorized into two cohorts, namely T2DM with IR group (292 cases) and T2DM without IR group (113 cases), as determined by a homeostasis model assessment-IR (HOMA-IR) value exceeding 2.0.

**Results:**

The findings of this study demonstrated compelling evidence of distinct biomarker profiles between individuals with T2DM who had IR and those without IR. Specifically, the IR individuals displayed notably raise NLR, MLR, PLR, C reactive protein (CRP) and serum amyloid A (SAA). Additionally, there was a noticeable decrease in superoxide dismutase (SOD) levels. Furthermore, IR was negatively correlated with SOD values, while positive associations were found between IR and NLR, CRP, and SAA levels (*p* < 0.05). Moreover, a rise in NLR and PLR levels demonstrated an identical relationship with the prevalence of IR (*p* = 0.007, *p* = 0.025, separately). The Receiver operating characteristic (ROC) curve demonstrated that the areas under the curve (AUC) for NLR, MLR, PLR, CRP, SAA and SOD in predicting occurrence of IR in T2DM patients were 0.603, 0.575, 0.581, 0.644, 0.594 and 0.632 respectively, with sensitivity of 79.5%, 95.2%, 46.9%,54.1% (or 51.4), 47.6% (or 45.7%) and 98.6% and specificity of 37.2%, 19.5%, 69.9%, 69% (or 71.7%), 71.6% (or 73.5%) and 23% respectively.

**Conclusion:**

Our findings support the notion that higher magnitude of NLR, PLR, MLR, CRP, and SAA values, corresponded to lower SOD levels, indicating a more severe degree of IR in T2DM patients. Additionally, NLR, PLR, MLR, CRP, SAA, and SOD demonstrated predictive potential for assessing IR. Regrettably, due to the retrospective nature of this study, it was not feasible to take a measurement the majority of inflammatory factors and reactive oxygen species (ROS).

## Introduction

Diabetes Mellitus (DM), 90% of which belongs to T2DM, and it has become a global epidemic non-communicable disease that threatens human life and health [1]. There were about 537 million diabetics in the world, and 6.7 million patients died of diabetes or its complications every year. China had the highest number of DM patients in the world, surpassing 116.4 million individuals [2]. IR and the progressive decline of islet β cell function was the main feature of T2DM. IR refered to the decreased sensitivity of peripheral tissues (mainly liver, fat and adipose tissues) to insulin action caused by various reasons, which reduced the efficiency of insulin in promoting glucose uptake and utilization. Upon the onset of IR, there is an elevation in oxidative stress (OS) within islet β cells and peripheral tissues, resulting in impaired insulin secretion, and the development of T2DM [3].

Individuals susceptible to T2DM were activated by various factors, and a variety of inflammatory factors resulted in IR, which caused the deterioration of patients’ condition [4]. T2DM patients were often accompanied by low-grade chronic inflammation [5]. SAA was a highly sensitive inflammatory marker discovered in recent years, and its expression in peripheral blood was significantly increased in acute and chronic inflammatory reactions [6]. According to a comprehensive meta-analysis, patients diagnosed with T2DM exhibited a marked elevation in SAA levels [7]. OS was the main defense system of the body, which could maintain normal physiological activities and balance the generation and elimination of reactive oxygen species (ROS) [8]. Once the above balance was broken, ROS would increase abnormally, so that the secretion of oxygen free radicals would increase, which would cause damage to the body, decrease the body’s defense ability, lack of antioxidant substances, and metabolic disorder, which would damage islet β cells, enhance IR, lack insulin secretion, increase blood sugar, and induce T2DM [9].

Therefore, chronic inflammatory reaction and OS may be the important causes of IR. Prolonged and chronic inflammation in the body resulted in an upregulation of inflammatory factors within the islet microenvironment, including interleukin-1β (IL-1β), CRP, tumor necrosis factor-α (TNF-α), which destroyed the function and activity of islet β cells, increased OS, and caused or aggravated IR in peripheral tissues [10]. NLR, MLR and PLR are recently proposed inflammatory markers. A large number of studies had found that NLR, MLR and PLR could predict diabetes and its chronic complications. In a study by Shiny [11], the relationship between NLR and varying extent of impaired glucose tolerance and IR was examined. The findings announced a positive pertinence between NLR and fasting blood glucose (FBG), HOMA-IR. Within T2DM, elevated levels of NLR and PLR had been found to be positively associated with microalbuminuria [12], as well as being relevant to severity of carotid plaque [13] and stable ischemic heart disease [14]. Among individuals with diabetic retinopathy (DR), there was a notable elevation in the levels of NLR, MLR and PLR in comparison to those without DR [15].

Currently, there was scarce literature available regarding the linkage between IR and NLR, MLR, PLR, CRP, SAA, and OS. The primary aim of this research was to investigate the association between these biomarkers and IR in individuals with T2DM, with the purpose of offering clinical treatment evidence.

## Methods

### Participants

In this study, a retrospective cross-sectional survey design was employed to investigate a cohort of 1025 patients diagnosed with T2DM during the period from January 2021 to November 2022. However, several participants were excluded based on specific criteria: 35 individuals with malignant tumors, 55 individuals with type 1 diabetes, 36 pregnant individuals, 84 individuals with acute diabetic complications, 68 individuals with acute attack of cardio-cerebrovascular illness, 102 individuals with serious liver and kidney dysfunction, 23 individuals with extremely abnormal white blood cell counts (> 50 × 10^9^/L or < 1.0 × 10^9^/L), and 56 and 161 individuals who received antibiotic treatment before or after admission, respectively. Ultimately, the study included 405 participants (comprising 274 men and 131 women) to analyze and draw conclusions.

### Data acquisition

The research team conducted a comprehensive data collection process, encompassing information on gender, age, height, weight, smoking experience (whether you quit smoking or not is regarded as smoking experience), past medical experiences. Blood pressure measurements were also recorded. Subsequently, fasting sufferers provided venous blood specimen following admission. Using an automated biochemical analyzer (7600-020; Hitachi),biochemical parameters were determined. Enzyme-linked immunosorbent assay (A2000 Plus; Autolumo) was employed to assess the levels of fasting C-peptide (FCP) and fasting insulin (FINS). Additionally, routine blood parameters were analyzed using an automated blood analyzer (Sysmex XN9000). CRP and SAA were detected by immunoturbidimetry. Detection of superoxide dismutase (SOD) by pyrogallol method.

### Definitions and calculation

In accordance with the 1999 guidelines provided by the World Health Organization (WHO), the study implemented standardized diagnostic criteria for identifying individuals with T2DM. To assess the body composition of participants, the study employed the body mass index (BMI) as a reliable metric. BMI was derived by dividing an individual’s body weight (in kilograms) by the square of their height (in meters). In order to evaluate the level of IR in the participants, the study employed the HOMA-IR. HOMA-IR was calculated using a formula that involved multiplying the FBG in millimoles per liter (mmol/L) by the FINS in microinternational units per milliliter (uIU/mL), and then dividing the result by 22.5. In conformity with HOMA-IR > 2.0, it was defined as IR [16]. The study derived the NLR, MLR, and PLR by dividing the respective counts of neutrophils, monocytes, and platelets by the count of lymphocytes.

### Statistical evaluation

The data evaluation was performed using SPSS 21.0. To evaluate the normality of the data, the study utilized the Kolmogorov-Smirnov test. For variables that exhibited a normal distribution, descriptive statistics including the mean ± standard deviation (SD) were employed to summarize the data. Tndependent T tests were utilized to compare the two groups. For continuous variables that deviated from a normal distribution, the study reported the medians along with interquartile intervals (IQR). To compare these variables between groups, the Mann-Whitney rank sum test was utilized. Categorical variables were reported as case numbers. To evaluate discrepancy within multiple groups, the chi-square test was employed. To investigate the association between other variables and IR, the study employed Spearman’s relation analysis. The study utilized logistic regression analysis to examine the associations between NLR, MLR, PLR and IR. A significance level of *P* < 0.05 or *P* < 0.01 was employed to determine statistical significance.

## Results

### Comparison of common survey results and lab test parameters

The prevalence of IR with T2DM was 292 patients (72.1%) (Table 1). In comparison to the non-IR group, individuals with IR demonstrated elevated levels of BMI, liver function parameters, glycolipid metabolism indexs (such as FBG, triglyceride (TG), total cholesterol (TC), low-density lipoprotein (LDL)), uric acid (UA), FCP, FINS, white blood cell (WBC), neutrophil, NLR, MLR, PLR and CRP. Conversely, In individuals with IR, there was a notable reduction in high-density lipoprotein (HDL) and SOD levels (*p* < 0.05 or *p* < 0.01). There were no notable distinctions observed between the two groups regarding gender, age, blood pressure, smoking experience, creatinine (CR), lymphocyte count, monocyte count, and platelet count.

**Table 1:** Comparison of common survey results and lab test parameters

	T2DM with IR (*n* = 292)	T2DM without IR (*n* = 113)	P
Gender(male/female)	193/99	81/32	0.281
Age(years)	56.68 ± 9.35	58.42 ± 9.26	0.092
Smoking^a^	84(28.8)	43(38.1)	0.071
BMI(kg/m^2^)	25.60 ± 2.85	23.45 ± 3.545	0.000
Systolic pressure(mmHg)	132.92 ± 15.81	134.02 ± 16.02	0.534
Diastolic pressure(mmHg)	82.33 ± 10.37	81.14 ± 9.35	0.288
GGT (U/L)	25.0(19.0–38.0)	19.0(14.0-23.5)	0.000
ALT(U/L)	20.0(14.6–28.3)	15.2(12.0–19.0)	0.000
AST(U/L)	19.0(15.0–23.0)	17.0(15.0-19.5)	0.003
TG (mmol/L)	1.96(1.35–2.92)	1.22(0.89–1.73)	0.000
TC(mmol/L)	4.89 ± 1.12	4.63 ± 0.97	0.035
HDL (mmol/L)	1.09(0.97–1.25)	1.18(1.06–1.38)	0.000
LDL (mmol/L)	3.06 ± 0.79	2.87 ± 0.68	0.029
CR(umol/L)	65.30(55.03–74.78)	65.90(59.45–76.95)	0.317
UA(umol/L)	341.01 ± 89.31	312.00 ± 78.61	0.003
FBG (mmol/L)	7.86(6.18–10.09)	6.10(5.34–7.19)	0.000
FCP (ng/mL)	2.46(1.77–3.16)	1.62(1.18–2.01)	0.000
FINS(uIU/ml)	11.69(8.31–17.12)	4.41(3.16–6.27)	0.000
HOMA-IR	3.96(2.82-6.00)	1380(0.95–1.85)	0.000
WBC(10^9^/L)	5.99(5.31–7.14)	5.65(4.84–6.57)	0.002
Neutrophil(10^9^/L)	3.28(2.75-4.00)	3.04(2.56–3.65)	0.014
Lymphocyte(10^9^/L)	2.06(1.70–2.49)	2.23(1.73–2.85)	0.052
Monocyte(10^9^/L)	0.49 ± 0.13	0.47 ± 0.11	0.184
Platelet(10^9^/L)	199.14 ± 50.40	191.87 ± 43.59	0.151
NLR	1.56(1.24-2.00)	1.41(1.05–1.75)	0.001
MLR	0.23(0.18–0.30)	0.21(0.15–0.27)	0.019
PLR	93.44(73.08-119.67)	83.86(64.93-108.13)	0.011
CRP(mg/L)	1.40(0.71–2.57)	0.86(0.46–1.58)	0.000
SOD(U/mL)	172.66 ± 18.32	184.22 ± 21.92	0.000

*Abbreviations* T2DM, type 2 diabetes mellitus; IR, insulin resistance; BMI, body mass index; GGT, glutamyltransferase; ALT, alanine aminotransferase; AST, aspartic acid aminotransferase; TG, triglyceride; TC, total cholesterol; HDL, high-density lipoprotein; LDL, low-density lipoprotein; CR, creatinine; UA, uric acid; FBG, fasting blood glucose; FCP, fasting C-peptide; FINS, fasting insulin; HOMA-IR, homeostasis model assessment for insulin resistance; WBC, white blood cells; NLR, rate of neutrophils to lymphocytes; MLR, rate of monocytes to lymphocytes; PLR, rate of platelets to lymphocytes. CRP,C-reactive protein; SOD,superoxide dismutase

Among 405 patients with T2DM, serum SAA levels were detected in 356 patients. These T2DM patients were split into the T2DM with IR group (164 men, 90 women) and the without IR group (75 men, 27 women). The level of HOMA-IR and SAA in T2DM with IR were higher than that in T2DM without IR (HOMA-IR: 4.00(2.84–6.21) vs. 1.33(0.92–1.82), *p* = 0.000; SAA: 3.25(1.63–4.95) mg/L vs. 2.48. (1.36–3.76) mg/L, *p* = 0.006).

### Correlation between IR and the other variables

Our findings revealed significant associations between IR and various variables (Table 2). Specifically, IR showed a negative correlation with gender, HDL, and SOD values, while displaying positive correlations with BMI, liver function parameters, glycolipid metabolism indexs (such as FBG, TG, TC, LDL), UA, WBC, neutrophil count, CRP, and NLR levels (*p* < 0.05 or *p* < 0.01). In a cohort of 356 patients diagnosed with T2DM who were assessed for SAA levels, a significant positive relation was observed between IR and SAA levels (*r* = 0.104, *p* = 0.049).

**Table 2:** Correlation between IR and the other variables

	r	P
Gender	-0.12	0.016
BMI	0.354	0.000
Systolic pressure	0.002	0.970
Diastolic pressure	0.063	0.204
GGT	0.414	0.000
ALT	0.355	0.000
AST	0.188	0.000
TG	0.449	0.000
TC	0.127	0.010
HDL	-0.215	0.000
LDL	0.12	0.016
CR	-0.041	0.415
UA	0.168	0.001
FBG	0.417	0.000
WBC	0.231	0.000
Neutrophil	0.185	0.000
Lymphocyte	-0.003	0.953
Monocyte	0.096	0.053
Platelet	0.044	0.382
NLR	0.129	0.009
MLR	0.054	0.275
PLR	0.037	0.456
CRP	0.263	0.000
SOD	-0.12	0.016

### The study employed logistic regression analysis to investigate the correlation between levels of NLR, MLR, PLR and the risk of IR

The study utilized multiple logistic regression analysis to investigate the impact of NLR, MLR, and PLR as independent variables on IR. Several other factors were considered as covariates. These factors included gender, smoking, BMI, ALT, TG, FBG, LDL, CRP, and SOD. The findings revealed significant independent positive associations between NLR, MLR, and PLR levels with IR (NLR: odds ratio (OR): 1.793, 95% confidence interval (CI): 1.115–2.885, *p* = 0.016); MLR: OR: 26.767, 95% CI: 1.091-656.945, *p* = 0.044; PLR (OR: 1.011, 95% CI: 1.002–1.019, *p* = 0.014). Moreover, our analysis identified BMI, ALT, TG, FBG, and SOD as significant contributing factors to IR in patients diagnosed with T2DM (Tables 3, 4 and 5).

**Table 3:** The study employed logistic regression analysis to investigate the correlation between levels of NLR and the risk of IR

	β	OR(95%CI)	p
Gender	0.511	1.666(0.840–3.306)	0.144
Smoking	0.670	1.955(1.006–3.798)	0.048
BMI	0.234	1.263(1.131–1.411)	0.000
ALT	0.077	1.080(1.042–1.119)	0.000
TG	0.309	1.362(1.086–1.708)	0.008
FBG	0.262	1.300(1.156–1.461)	0.000
LDL	0.145	1.156(0.777–1.720)	0.474
NLR	0.584	1.793(1.115–2.885)	0.016
CRP	-0.027	0.974(0.867–1.094)	0.654
SOD	-0.042	0.959(0.945–0.974)	0.000

**Table 4:** The study employed logistic regression analysis to investigate the correlation between levels of MLR and the risk of IR

	β	OR(95%CI)	p
Gender	0.788	2.198(1.192–4.054)	0.012
BMI	0.235	1.265(1.134–1.411)	0.000
ALT	0.073	1.076(1.039–1.114)	0.000
TG	0.404	1.498(1.173–1.912)	0.001
FBG	0.255	1.290(1.149–1.448)	0.000
LDL	0.070	1.073(0.733–1.570)	0.717
MLR	3.287	26.767(1.091-656.945)	0.044
CRP	-0.009	0.991(0.884–1.111)	0.875
SOD	-0.041	0.960(0.945–0.974)	0.000

**Table 5:** The study employed logistic regression analysis to investigate the correlation between levels of PLR and the risk of IR

	β	OR(95%CI)	p
Gender	0.441	1.555(0.788–3.069)	0.203
Smoking	0.622	1.863(0.957–3.628)	0.067
BMI	0.236	1.266(1.133–1.415)	0.000
ALT	0.077	1.080(1.043–1.120)	0.000
TG	0.448	1.565(1.218–2.013)	0.000
FBG	0.254	1.289(1.146–1.449)	0.000
LDL	0.070	1.073(0.730–1.575)	0.721
PLR	0.011	1.011(1.002–1.019)	0.014
CRP	-0.013	0.987(0.878–1.110)	0.826
SOD	-0.043	0.957(0.943–0.972)	0.000

### Relationship between the levels of NLR, MLR and PLR with the prevalence of IR

To investigate the association between NLR, MLR, PLR levels and occurrence of IR, we consolidated all patients into a unified cohort for analysis. The three-point numbers of NLR were T1<1.29 (*n* = 135 patients), 1.29 ≤ T2 < 1.77 (*n* = 135 patients), T3 ≥ 1.77(*n* = 135 patients). The three-point numbers of MLR were T1<0.19 (*n* = 135 patients), 0.19 ≤ T2 < 0.26 (*n* = 135 patients), T3 ≥ 0.26 (*n* = 135 patients). The three-point numbers of PLR were T1<78.01 (*n* = 135 patients), 78.01 ≤ T2 < 106.87(*n* = 135 patients), T3 ≥ 106.87 (*n* = 135 patients). There was an progressive rise in the prevalence of IR as the trivalent of NLR and PLR increased (T1: 63%, T2: 73.3%, T3: 80%, *p* = 0.007 in NLR; T1: 64.4%, T2: 72.6%, T3: 79.3%, *p* = 0.025 in PLR). Nevertheless, no significant variation was found in the prevalence of IR as the levels of MLR increased.

### NLR, MLR, PLR, CRP, SOD and SAA predicted the risk of IR

To evaluate the predictive performance of the variables, including NLR, MLR, PLR, CRP, SOD, and SAA, their impact on predicting IR risk was assessed using the ROC curve. The study findings revealed that NLR, MLR, PLR, CRP, SOD, and SAA had significant predictive value in assessing the risk of IR among patients with T2DM. The AUC values and their corresponding 95% CI were as follows: NLR: AUC = 0.603, 95% CI 0.541∼0.665, *P* = 0.001; MLR: AUC = 0.575, 95% CI 0.511∼0.639, *P* = 0.019; PLR: AUC = 0.581, 95% CI 0.518∼0.645, *P* = 0.011; CRP: AUC = 0.644, 95% CI 0.584∼0.705, *P* = 0.000; SOD: AUC = 0.632, 95% CI 0.571∼0.693, *P* = 0.000; SAA: AUC = 0.594, 95% CI 0.529∼0.658, *P* = 0.006, respectively. The optimal cut-off point determined to be NLR = 0.167, with a sensitivity of 79.5% and a specificity of 37.2%; MLR = 0.147, with a sensitivity of 95.2% and a specificity of 19.5%; PLR = 0.168, with a sensitivity of 46.9% and a specificity of 69.9%; CRP = 0.231, with sensitivity of 54.1% or 51.4% and corresponding specificity of 69% or 71.7%; SOD = 0.216, with a sensitivity of 98.6% and a specificity of 23%; SAA = 0.192, with sensitivity of 47.6% or 45.7% and corresponding specificity of 71.6% or 73.5%.

## Discussion

T2DM represents a pressing issue in terms of global population health., and the low treatment rate and compliance rate of diabetic patients also aggravate the occurrence and progress of chronic complications of diabetic patients. IR and/or islet β cell dysfunction were the main causes of T2DM. The results of our study unveiled notable disparities in NLR, MLR, PLR, CRP, SAA, and SOD levels when comparing T2DM patients with IR to those without IR. Specifically, individuals with IR displayed elevated NLR, MLR, PLR, CRP, SAA levels, while SOD levels were prominently lower.

The development of T2DM involved intricate mechanisms. The occurrence of low-grade chronic inflammation in individuals with T2DM triggered an excessive secretion of inflammatory markers, including CRP, interleukin-6 (IL-6), TNF-α and monocyte chemokine 1(MCP-1). This inflammatory milieu contributed to an elevation in the number of neutrophils [17]. NLR was an emerging marker of inflammation. It represented the balance between neutrophils in non-specific inflammatory mediators and lymphocytes with inflammatory regulation or protection components. NLR was also a well-standardized and inexpensive measurement method, and it was more stable than a single neutrophil or lymphocyte [18]. Tong et al. [19] pointed out that there was a correlation between leukocytosis and chronic complications of diabetes, and the increase of leukocytes mainly reflected the rise of neutrophils in the body. When inflammation occured in the body, white blood cells responded quickly to inflammatory stimuli, resulting in neutrophil increase in the circulation. On the other hand, the increase of interleukin levels could promote the decrease of lymphocytes and the increase of neutrophils [20, 21], which led to the increase of NLR and the continuous increase of inflammation levels in the body, thus mediating IR [22]. NLR was increased in diabetes and IR. Duman [23] discovered that individuals diagnosed T2DM exhibited markedly elevated NLR levels and WBC count in comparison to healthy individuals. Furthermore, NLR was found to be closely associated with FBG and glycosylated hemoglobin (HbA1c) levels. Lou [24] conducted a study that revealed higher levels of NLR in individuals with T2DM who had IR compared to those without IR. An elevated NLR could serve as a dependable indicator of IR. Our study findings aligned closely with those of Lou, as we observed a consistent positive association between the risk of IR and NLR in individuals with T2DM. Notably, as NLR levels increased, there was a significant upward trend in the prevalence of IR. Moreover, our analysis revealed a robust predictive capacity of NLR in identifying IR.

The presence of inflammation was assessed by using an elevated monocyte count and a reduced lymphocyte count as indicators [25]. MCP-1 secreted by monocytes and macrophages could promote inflammatory cells to gather at the lesion site, thus stimulating monocytes to secrete IL-1 and IL-6, making pancreatic tissue in a state of micro-inflammation, damaging endothelial cells, raising blood sugar, generating oxidative stress, triggering IR [26]. The research conducted by Kocak MZ [27] revealed that individuals diagnosed 2DM and microalbuminuria displayed significantly elevated MLR levels. Furthermore, MLR showed a positive correlation with the presence of microalbuminuria.Importantly, the study identified MLR, FBG, and HbA1c as independent predictors of diabetic renal damage. Yue et al. [28] also proved that MLR was a risk factor for DR.

Platelets could participate in the inflammatory response of the body by releasing proinflammatory factors to recruit and activate white blood cells [29]. In individuals with diabetes, platelets exhibited heightened activity, leading to the release of inflammatory mediators that attracted additional platelets and WBC to the site of inflammation [30]. Conversely, the heightened presence of ROS contribute to apoptosis or programmed cell death in lymphocytes. This process may ultimately result in a reduction in lymphocyte count [31]. In the investigation conducted by Zhang [32], notable disparities were observed between individuals with diabetic foot and those without foot complications. Specifically, the study unveiled a significant elevation in platelet counts among diabetic foot individuals, while concurrently demonstrating a noteworthy reduction in lymphocyte counts. Moreover, the researchers identified a positive association between the Wagner classification of diabetic foot and IR. This suggested that PLR could serve as a valuable marker for assessing the extent of diabetic foot complications. The above-mentioned studies on MLR and PLR were conducted among people with diabetes complicated with chronic complications. We studied people with diabetes complicated with IR.

In our investigation, we observed a significant increase in both MLR and PLR in individuals with T2DM who had IR. These findings indicated that higher MLR and PLR values were related to the rising risk of IR. Furthermore, MLR and PLR demonstrated a certain degree of predictive value for identifying IR in this patient population. This was the first report on the connection between MLR, PLR and IR in T2DM with IR.

CRP, as a non-glycosylated polyprotein, its biological activity was mainly manifested in binding endogenous foreign bodies, activating complement and regulating phagocytic activity at the same time, which rose rapidly in inflammatory reaction and was a major inflammatory factor in the body [33]. SAA was a local and systemic pro-inflammatory cytokine, which was mainly synthesized by hepatocytes and adipose tissue cells. SAA served as a prominent acute phase protein in both humans and mammals [34]. Its presence could elicit the upregulation of CRP and interleukin-6 (IL-6) production [35]. These inflammatory factors, in turn, had the ability to hinder the signal transduction pathway of insulin action. Consequently, this disruption led to the development of IR at both localized and systemic levels in affected patients [36]. Liu T [7] discovered that individuals diagnosed T2DM showcased rised SAA levels. Importantly, the findings indicated a positive relevant between SAA levels and HOMA-IR, CRP, IL-6. SAA levels were also increased in the mouse model of diabetic stroke, which were the key factor for the progress of T2DM [37]. Our research clearly demonstrated that patients diagnosed T2DM who had IR displayed elevatory CRP and SAA levels. Notably, these elevated levels of CRP and SAA demonstrated a positive association with the presence of IR. These findings suggested that the inflammatory response mediated by CRP and SAA actively contributed to the pathophysiological processes involved in T2DM. And the more serious the inflammatory reaction, the more obvious the degree of IR, which also had certain predictive value for IR in T2DM patients. This was consistent with the above research conclusions. In patients with T2DM, insulin was relatively deficient, and insulin sensitivity decreased. The high glucose environment feedback regulated the glucose metabolism of liver and adipose tissue. SAA may participate in and mediate this process, but with the progress of the disease, IR wouldl further increase, which would induce the waterfall effect of inflammation, and excessive SAA would aggravate the inflammatory damage of tissues and organs.

Pancreatic β -cell damage caused by OS was an important mechanism for the occurrence and development of diabetes [38]. Due to the influence of hyperglycemia environment, the metabolism of glucose and lipid in DM patients was disordered, which would release a large number of oxygen free radicals, inhibit the ability of free radical scavenging in a feedback way, resulting in imbalance of antioxidant function, enhanced OS, increased levels of ROS, oxidative damage of islet β cells, resulting in impaired function, enhanced IR and induced T2DM [39]. Sod, as an antioxidant, was the first line of defense against OS, which could specifically scavenge superoxide anion free radicals [40]. Liu J et al [41] observed that compared with healthy mice, the levels of ROS in β-cells of T2DM mice increased and the levels of SOD decreased, which indicated that OS was induced in T2DM. The study conducted by Zhang G [42], yielded similar findings, revealing a notable decrease in SOD concentration in individuals diagnosed with T2DM. Importantly, this decrease in SOD concentration was found to be negatively correlated with the presence of IR. In our investigation, we discovered a significant decrease in SOD levels among individuals diagnosed with T2DM who had IR. Importantly, this decrease in SOD levels demonstrated a negative correlation with the presence of IR. Notably, our study also revealed that SOD levels held certain predictive value for identifying IR. Our findings indicated a clear association between inflammatory response, OS reaction, and IR in individuals diagnosed with T2DM.

IR was often characterized by dyslipidemia, including the increase of TG, LDL-C and free fatty acid levels, and the decrease of HDL-C levels [43]. In our study, we discovered significant increases in BMI, glycolipid metabolism indexs (such as FBG, TG, TC, LDL) among individuals diagnosed with T2DM who had IR. Conversely, we observed a significant decrease in HDL levels in these patients. Our findings demonstrated a noteworthy positive associations between IR and BMI, glycolipid metabolism indexs (such as FBG, TG, TC, LDL). In contrast, the negative correlation between IR and HDL. Even after accounting for potential confounding variables, such as BMI, TG, FBG and LDL, our study consistently revealed that NLR, MLR and PLR remained significant risk factors for developing T2DM. This may be because obesity and high fat intake could promote the intake of fatty acids and increase fatty acid metabolites in muscle cells, aggravate the inflammatory response of the body, interfere with insulin signaling pathway, raise blood sugar and aggravate IR. Duffy et al. [44] investigated that among smokers, the levels of NLR and PLR were relatively high, and NLR was related to smoking intensity. Upon evaluating the association between smoking and IR in our study, we observed no statistically significant disparity in smoking prevalence between the group of individuals diagnosed with T2DM and IR and the group without IR. After correcting smoking, NLR and PLR were still found to be positively correlated with IR.

Our research had that follow limitations: First, Given the restricted sample size in the research, we were unable to examine the impact of several factors, including existence of chronic complications, as well as the use of hypoglycemic and lipid-regulating medications, it was imperative to conduct further research with a more robust design that incorporated stratified analysis. Second, it was essential to acknowledge that our study design was retrospective and cross-sectional in nature, which limits our ability to establish a definitive causality between NLR, MLR, PLR, CRP, SAA, SOD and IR. Thirdly, given the retrospective nature of this study, the measurement of numerous inflammatory markers and ROS was not impracticable, which may increase the accuracy of the results. However, related cross-sectional and prospective studies had shown thatT2DM and its complications were positively correlated with IL-6, TNF-α and malondialdehyde (MDA) [45, 46]. In line with previous research conducted by Kahraman C and colleagues [47], the study also demonstrated a positive relation between NLR and IL-6. Therefore, we chose inflammatory markers that could be widely used in clinic and standardized in measurement. Finally, due to the variability of blood routine parameters in different periods, we only chose results of a blood draw and drew a conclusion that there may be some differences. If the average method was used for multiple tests of indicators, the reliability of the results would be increased.

## Conclusions

In summary, NLR, PLR and MLR were cheap, standardized and easily available inflammatory markers, CRP, SAA and SOD were also indicators of routine clinical detection, which had certain clinical value in predicting IR in patients with T2DM.

## Data Availability

The material utilized in this manuscript can be obtained by contacting the corresponding writer. However, it was important to note that the release of data was contingent upon the publication of the findings.
